# Mortality and morbidity in ageing men: Biology, Lifestyle and Environment

**DOI:** 10.1007/s11154-022-09737-6

**Published:** 2022-06-14

**Authors:** Erfei Zhao, Eileen M. Crimmins

**Affiliations:** Davis School of Gerontology, University of Southern California, 90089-0191 Los Angeles, CA United States

**Keywords:** Sex, Gender, Life Expectancy, Heart Disease, Stroke, Diabetes, Hypertension, Cholesterol, Epigenetic aging

## Abstract

Males live shorter lives than women in all countries. The universality of shorter male life expectancy is a 21st Century phenomena. It occurs with the decline in infectious diseases and the rise in cardiovascular diseases accounting for mortality. Male/female differences in morbidity are not as succinctly characterized. Men have a higher prevalence of lethal diseases, which is linked to their lower life expectancy. Women have more non-lethal conditions such as depression and arthritis; which may also be linked in part to longer survival. Men have better physical functioning and less disability which is partly explained by gender differences in diseases and also by their greater strength, size, and stamina. Gender differences in risk factors for disease have changed over time with the prevalence and treatment of risk as well as differential behavior by gender. Examination of what are seen as basic molecular and cellular measures related to aging indicates men age faster than women; however, even these basic biological measures result from a combination of biology, behavior, and social factors.

## Introduction

Mortality of men in all age groups is higher than that of women; in no country does the life expectancy of men exceed that of women [1]. Men have a higher prevalence of a number of diseases that are important in determining life expectancy; on the other hand, men in most countries have fewer problems with physical functioning than women [2]. What are the reasons for the differences – are they biological, do they result from behavioral differences, or do they reflect the varying social and epidemiological environments in which men and women live? Does male sex, as compared to female, predispose men to certain diseases and to mortality from certain causes, and are there diseases in which male sex is protective? By what means could the morbidity and mortality gaps between men and women be narrowed?

Our review will begin with a discussion of the model of the morbidity process in order to clarify how introducing dimensions of health can add to our understanding of the differences by sex or gender. In earlier work we have rejected the conclusion that men have higher mortality but better health than women, indicating that this is too simplistic a conclusion [2]. Our conceptualization of health as a process of change with age by which different dimensions of health are affected helps understand this complexity. We then discuss empirically observed sex differences in mortality, physical functioning, cognitive functioning, major diseases, and biological and molecular and cellular mechanisms of aging that reflect risk for the downstream outcomes in the model. As we discuss each major aspect of the morbidity process, we will indicate what we know about differentials across countries in order to integrate environmental context into our discussion of male/female differences. We also indicate the biological and social factors that underlie these differences.

## The morbidity process

**Fig. 1:**
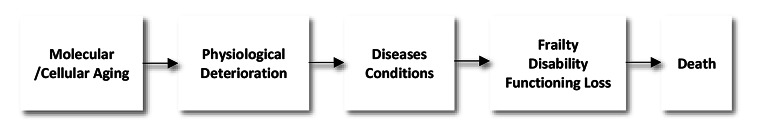
Health Change with Age - The morbidity process. Dimensions indicate time pattern of age-related health change at the population level. Updated version of Crimmins et al. [3]

To examine the excess mortality among males and sex differences in aspects of morbidity, our analysis uses an updated model of the morbidity process reflecting population health change with age that allows us to organize health change into its dimensions (Fig. 1). This model categorizes multiple health indicators into dimensions that describe the process of age-related health change at a population rather than individual level [3]. Specifically, age-related health change first starts with molecular and cellular aging, followed by physiological dysregulation indicated by various biological risk markers, such as increased blood pressure and increasing concentrations of total cholesterol; these changes then lead to increases in the development of physical and mental conditions, which are then followed by subsequent diagnosis of diseases, functioning loss and disability, frailty, and, ultimately, death. Any individual may have a different ordering of the process - they may even skip some of the dimensions or experience reversals in the process – but the process describes change in populations. It is also true that the links between the dimensions can change over time and differ across place. For instance, multiple dimensions of health can relate to mortality or upstream risk markers differently. For instance, health care interventions may reduce the links between disease and death which might change the sex differential in mortality and/or disease.

In order to better understand excess male mortality and differential morbidity, we examine sex differences across time and place in all dimensions of health to see whether the male morbidity/mortality excess is consistent across dimensions, over decades and across regions of the world. We hypothesize that differentials between male and female health differ across dimensions of health, over historical periods and between countries. We hypothesize that sex differences in multiple aspects of health allow few clear generalizations because they are rooted in behavior, social culture, epidemiology as well as biology.

## Mortality

**Fig. 2:**
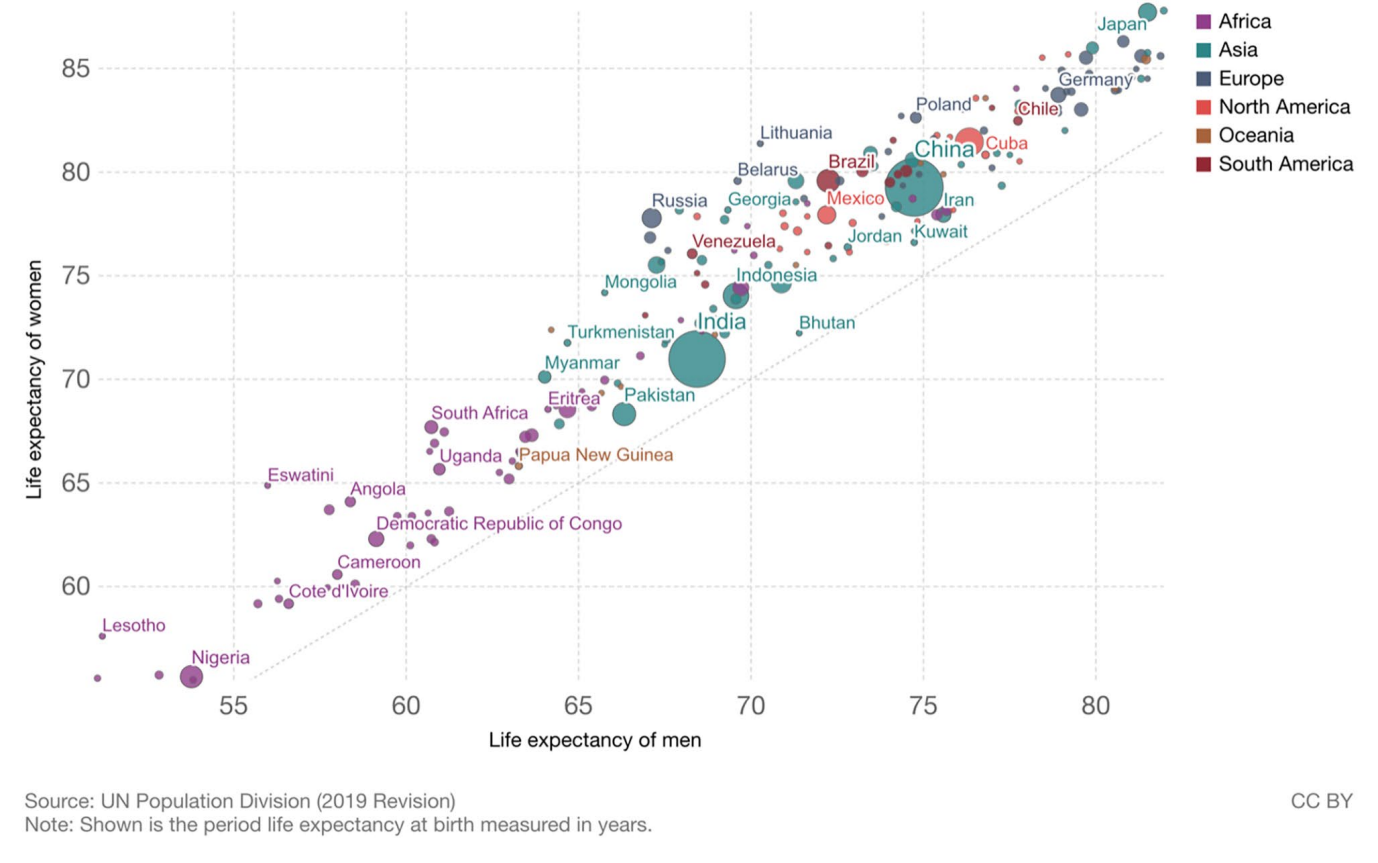
**Life expectancy of women vs. life expectancy of men 2019.** Source: United Nations, Department of Economic and Social Affairs, Population Division (2019). World Population Prospects: The 2019 Revision, DVD Edition. Max Roser, Esteban Ortiz-Ospina and Hannah Ritchie (2013) - “Life Expectancy”. Published online at OurWorldInData.org. Retrieved from: ‘https://ourworldindata.org/life-expectancy’ [4]

As indicated above, male life expectancy is now lower than female life expectancy in all countries. Figure 2 shows that when male and female life expectancy are graphed with male life expectancy on the x axis and female on the y axis, all countries fall above the line of equality, indicating higher life expectancy for women [4]. These is clearly variability in the size of the differential with relatively longer female life expectancy in some African and Eastern European countries. Eastern European countries have large behavioral differences between men and women with drinking and smoking being major contributors [5]. So, while excess male mortality over the life span is higher and the resulting length of male life expectancy is lower than women’s in all countries, the size of the difference is not a constant.

There are examples of longer male life expectancy in some countries before 2006 when longer female life expectancy in all countries was established [1]. In 2005 there were still four countries in which women lived shorter lives than men - Niger women could expect to live a dozen days less than men and in Botswana three dozen days less, but nearer to two years less in Zimbabwe and Kenya [1]. Most examples of women having shorter lives than men are in societies where women bear many children without the help of modern medicine and where cigarette smoking and alcoholism are not yet important causes of death for men. This characterizes currently poor countries and the history of rich countries.

**Fig. 3:**
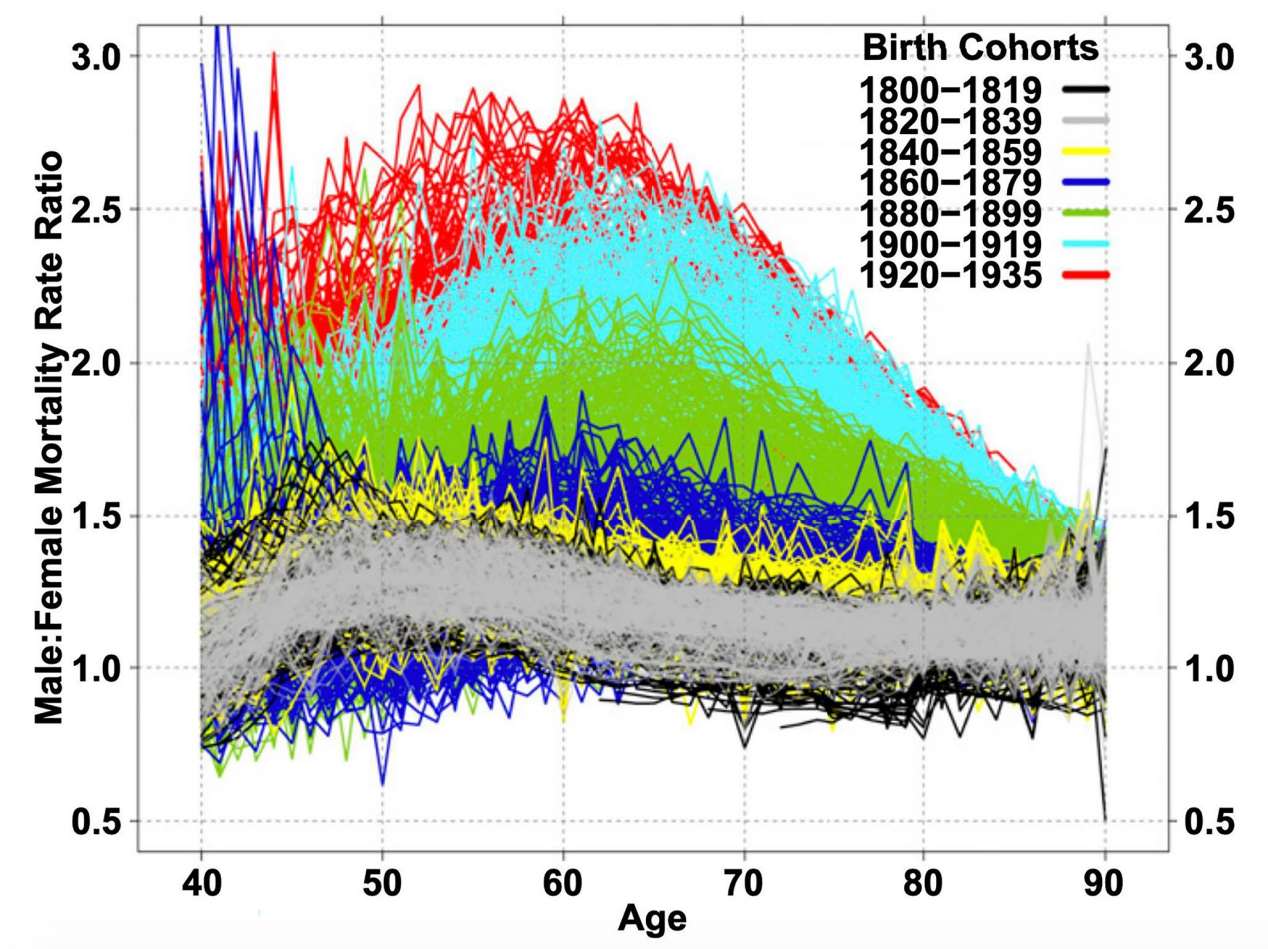
Male-Female mortality ratios for single-year birth cohorts for 13 countries: 1800–1935 [6]

The gender gap in mortality rates has changed markedly throughout the 20th century in countries where this can be observed. Examining changes over time in sex differences in mortality can give us some insight into the behavioral and epidemiological factors that might explain these differences. Examination of age-specific changes in male/female mortality ratios from age 40 onward for birth cohorts spanning the 135 years from 1800 to 1935 for thirteen countries shows that in the early part of this time period, male excess mortality was relatively small (as the ratio is just above 1.0) and that it has increased as time has gone on especially at ages forty through seventy (where it increased to 2.5 to 3.0) (Fig. 3). These large differences in male and female mortality ratios above age 40, occurring among those born in the 20th century, is the major explanation for the large discrepancies between male and female life expectancy in the latter part of the 20th century [6].

This increase in male/female mortality ratios in the last century is attributed to both the epidemiological environment and social and behavioral differences between the genders. Part of the difference between countries and the change over time results from epidemiological conditions reflecting differences in disease dominance. At the beginning of the 20th century, infectious diseases remained major causes of mortality and mortality rates of women and men were more similar. As infectious disease mortality among adults was replaced by chronic disease mortality as major causes of death, particularly cardiovascular conditions and cancers, the sex differential in mortality changed.

However, the change in the relative level of mortality rates for men and women does not only reflect epidemiological changes resulting in changes in causes of death overtime but also changes in risk-related behaviors for men and women [7–9]. Waldron [10] suggests that the largest differences between male and female death rates occur in causes of death (e.g., coronary heart disease, accidents, suicide, lung cancer, and cirrhosis of the liver) that are linked to behaviors encouraged or more accepted in males (e.g., Type A behavior, cigarette smoking, alcohol consumption, and working at hazardous jobs). Smoking among males seems to largely explain the initial surge in the male mortality excess, in which heart disease is the main condition associated with increased excess male mortality for those born after 1900 and smoking-attributable deaths account for about 30% of excess male mortality at ages 50–70 for cohorts born in 1900–1935 [6].

Nevertheless, in the most recent years, some countries have seen a decrease in the female advantage in life expectancy due to decreases in the male-female mortality ratio in the past two decades [11–13]. While some studies suggest that the recent reduction in the male mortality excess may be a result of relative improvements in men’s health, lifestyle or occupational environments, others attribute it to a worsening of the behaviors and circumstances of women. Women’s smoking behavior spread somewhat later than that of men and the effects of past smoking are now resulting in higher mortality from lung cancer, cardiovascular and others disease among women. The effects of past smoking had already been experienced by men. It is also thought that the changing societal roles of women have resulted in more stressful life circumstances reducing their mortality decline relative to men. In countries in which women and men are socially and behaviorally more equal, the slowing of female advantage in life expectancy is more apparent [14, 15].

It would be easy to attribute the rise in relative male mortality solely to behavioral and epidemiological change but study of male mortality excess in infancy indicates the explanation may be more complicated. A similar analysis of the sex ratio of infant mortality in 15 countries during the period from 1751 to 2004 reported marked change over time in the sex ratio of mortality among infants, an age when the effect of behavioral differences should be minimal. As infant mortality rates declined over 2 centuries from high to low, the excess of male infant mortality rates increased from only 10% in 1751 to more than 30% by approximately 1970; since 1970, the male disadvantage in most countries fell back to lower levels. These changes are attributed to both the reduced importance of infectious disease for male and female infants and improvement in obstetrical and neonatal care which appeared to benefit female infants more than males. Male infants’ greater biological weakness to both infectious diseases, perinatal problems, and other conditions associated with prematurity and development is the general explanation for higher male mortality in infancy [10]. Of course, cultural conditions can also play a role in gender differences in mortality among infants. In some societies in which the cultural norm values infant boys over girls, mortality among girls in infancy and early childhood, and missing female births may be higher than that of boys and reflect parental preference for males [16, 17].

Sex chromosomes may play a role in the sex differential in life expectancy. It is believed that the heterogametic sex, which refers to any organism with differing sex chromosomes (e.g., XY in male humans), may be more likely to express recessive deleterious mutation on the X chromosome which may in turn lead to decreased longevity. A recent meta-analysis reveals that the homogametic sex (e.g., XX in female humans), on average, lives 17.6% longer than the heterogametic sex [18]. Another clear example of excess male mortality influenced by biology is that castration seems to have a protective effect on male survival, which indicates that testosterone may be a determinant of the gender gap in human lifespan [19]. Specifically, historically, eunuchs castrated before the onset of puberty extended their mean life expectancies by 11 years; modern eunuchs castrated at similar age might expect to extend their life expectancies by 7 years. This may be the case that some of life expectancy gains from castration are due to the increased ability of eunuchs to fight off infections, and thus the gain in life expectancy among modern-day eunuchs is less pronounced as fewer men die from infections in the 21st century.

## Frailty, disability, and functioning loss

Before death, many people experience major health problems due to loss of physical functioning ability which can result in disability or an inability to do the tasks needed for self-care and independent living. These abilities have typically been measured in surveys with responses to questions on ability to perform specific tasks or to perform specified physical tasks. Ability to perform activities of daily living (ADL) are seen as necessary for self care. These functions include walking across a room, getting in and out of bed, bathing or showering, eating (such as cutting up your food), dressing (including putting on shoes and socks) and using the toilet (including getting up or down). Instrumental activities of daily living (IADL) are abilities needed for independent living which includes cognitive ability as well as physical ability. These include using a map to figure out how to get around in a strange place, preparing a hot meal, shopping for groceries, making telephone calls, taking medications, managing money, such as paying bills and keeping track of expenses. Additional physical functioning tasks regarded as abilities necessary for work including tasks related to mobility, strength, fine motor and endurance (e.g., walking, climbing stairs, getting up from a chair, lifting objects, picking up a coin, etc.).

**Fig. 4:**
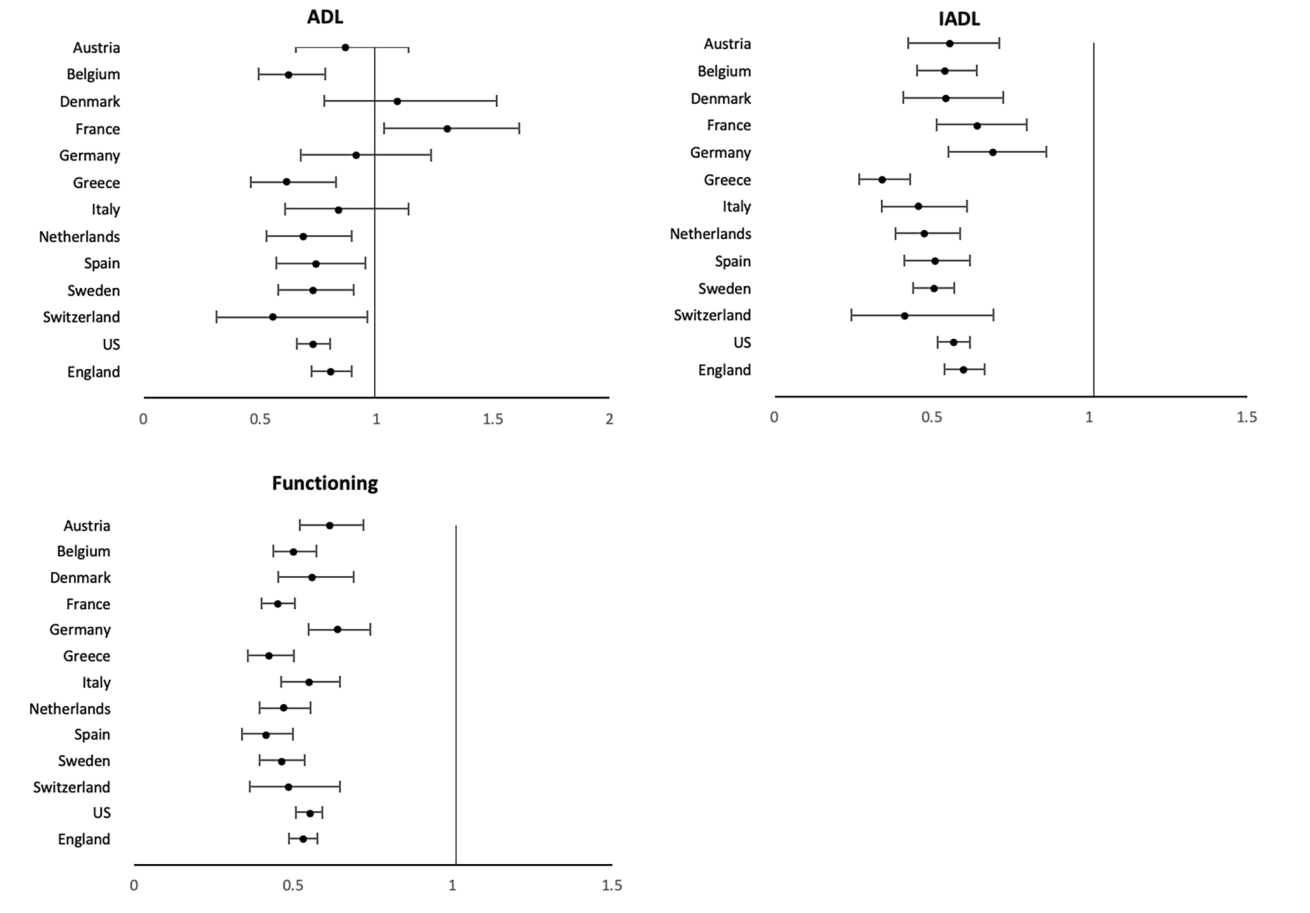
Odds Ratios indicating the effect of being male on ADL, IADL, and functioning difficulties for older populations in 13 countries. Odds ratios from logistic regressions of sex on the presence of condition when age controlled; vertical line indicates equality for men and women. U.S. data come from the Health and Retirement Study (HRS), England data come from the English Longitudinal Study of Ageing (ELSA), and other countries are from the Survey of Health, Ageing and Retirement in Europe (SHARE) [21]

For most of these activities in most countries men are more resilient against disability than women in the older ages [20]. The odds ratios indicating less difficulty of men relative to women in performing ADL, IADL and physical functioning tasks in 13 countries (Austria, Belgium, Denmark, France, Germany, Greece, Italy, the Netherlands, Spain, Sweden, Switzerland, England, and the US) are shown in Fig. 4. In all countries, men have a significantly lower chance than women of having IADL and physical functioning problems Regarding ADL difficulties, though men perform better than women on average, the gender difference is not as stark and is only significant in 8 countries. In France, more men than women actually reported difficulty in ADLs. This suggests that men have less physical disability than women overall, and that the gender gap is more pronounced when the tasks require more complex abilities compared to the more basic tasks such as bathing, dressing, eating, etc. [21]

There is growing recognition that sex inequalities in physical functioning probably start emerging in mid-life [22, 23] with women having an earlier age at onset of disability compared to men [24, 25].

There has been popular belief that the men’s resilience against disability may be more “social” than physiological [26]. Specifically, researchers suggested that, from an early age, men are generally taught to be tough and tolerate pain, whereas women are encouraged to be sensitive and to verbalize discomfort [27]. As most ADL and IADL data are self-reports, it is possible that men under-report difficulties of accomplishing certain tasks. However, Crimmins et al. found that when controls for the presences of diseases were included in the analysis, the sex differences in disability disappeared, suggesting that the worse functioning of women was explained by having more conditions that affect functioning rather than differential reporting [20]. Wheaton and Crimmins further addressed potential bias from self-reports by examining sex differences in performance measures of functioning, including gait speed, grip strength, and indicators of balance (tandem stand) and mobility (chair stand) [28]. Results showed that in performance tests men were more mobile and had better balance reflecting differences in men and women’s disability without reporting bias.

This female disadvantage in physical functioning is at least partly explained by the differences in body composition between men and women, in which women of all ages generally have lower muscle mass and strength [29, 30]. The higher testosterone levels among men than women may play a part in explaining this difference. Studies have generally shown that both endogenous and exogenous testosterone levels are modestly associated with muscle strength [31]. Men with hypogonadism due to androgen deprivation therapy have also been reported to have decreased strength [32]. Still, the association between testosterone level and physical function remain inconclusive, with some studies indicating that higher testosterone may be associated with better functioning measures such as handgrip and walking, while others indicating no improvement in strength or functional mobility [31].

It is also likely that women have worse physical functioning than men due to their weaker bones, especially among older adults. The bones of humans typically reach peak mass in their third or fourth decade of life; thereafter, men lose bone density at a steady pace, whereas women lose bone density at an accelerated rate for about 10 years after menopause, resulting in a much higher risk of osteoporosis among women than men [33]. Some of this is explained by social factors, as women take on less weight-bearing jobs or exercises and have lower levels of sun exposure compared to men [34]; biologically, estrogen loss at menopause and pregnancy and lactation, which divert calcium from the mother’s bones to the child, may both potentially lead to weaker bones and therefore worse physical functioning among women [35, 36]. Another possible explanation for some of the sex difference in physical function may be that the lower testosterone level among women is associated with lower hemoglobin concentrations, which is believed to be related to impaired physical function [37]. There are also social explanations for the gender difference in strength and body composition.

## Diseases and Conditions

**Fig. 5:**
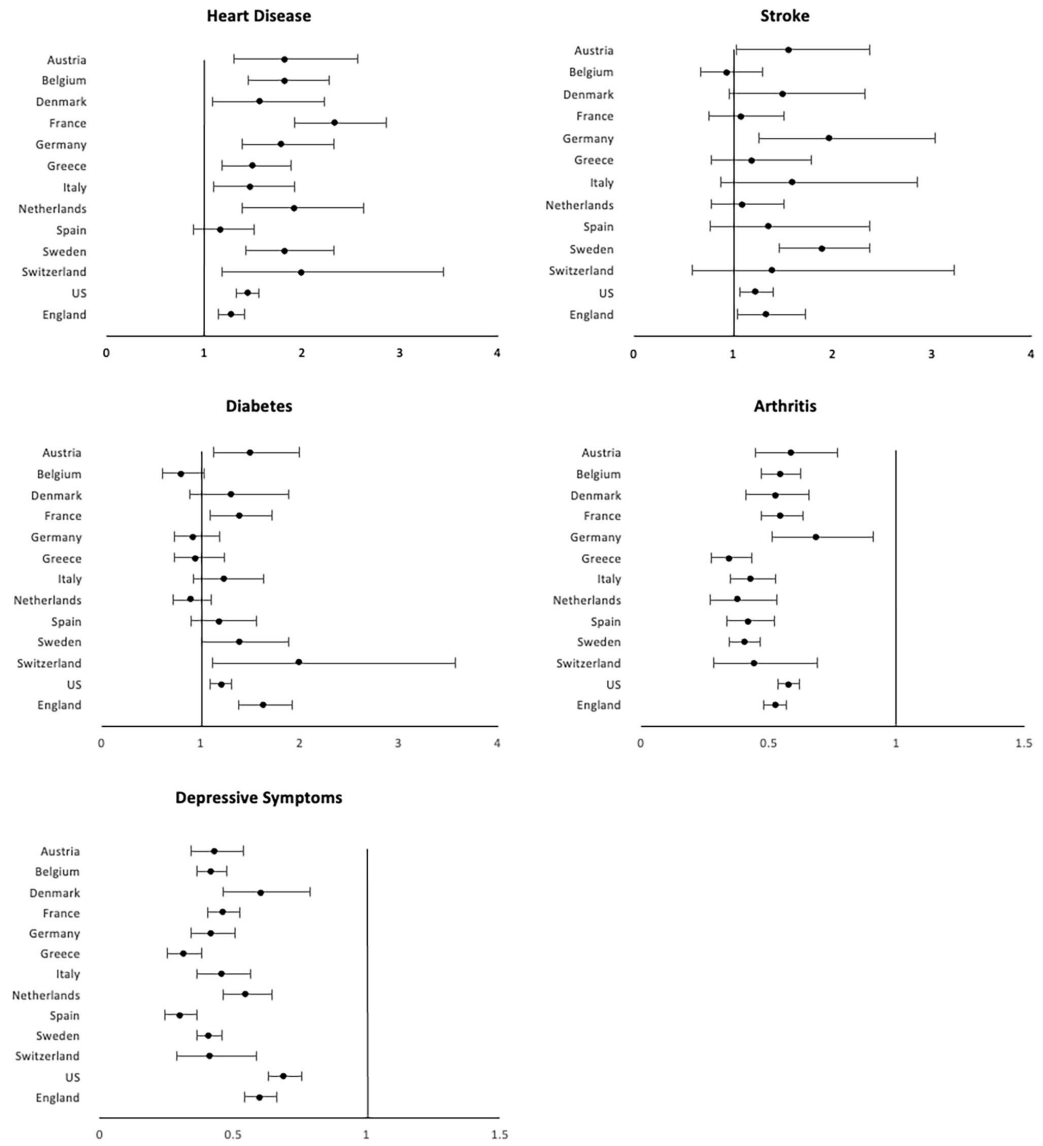
Odds ratios indicating effect of being male on presence of disease or condition among the older population (age ≥ 50 years). Vertical line indicates equality for men and women. U.S. data come from the Health and Retirement Study (HRS), England data come from the English Longitudinal Study of Ageing (ELSA), and other countries are from the Survey of Health, Ageing and Retirement in Europe (SHARE) [21]

Sex differences in the prevalence of diseases and conditions generally indicate that men have a higher prevalence of more lethal conditions, whereas women are more likely to have chronic but non-fatal diseases [2]. Figure 5 shows the male relative risk for major chronic conditions among people over 50 years of age in 13 countries. The observed conditions include heart disease, stroke, diabetes, arthritis, and depression. Men are generally more likely to report heart disease, stroke, and diabetes, whereas women are more likely to have arthritis and depression, though there is considerable variation between countries in the difference between the sexes.

In 12 out the 13 countries examined, men were significantly more likely to report having heart diseases. This higher level of heart disease in men is thought to arise from an earlier onset, by 5–10 years, as well as higher risk of developing heart diseases throughout the life course, though this gap gradually declines with age [38, 39]. There are multiple explanations of the excess male risk in heart disease. For instance, endogenous estrogen is believed to be cardioprotective among women through menopause; however, evidence of only minor changes in age-adjusted incidence of heart disease among women when moving from pre- to postmenopausal age does not provide strong support for this explanation [40].

Interestingly, although the higher testosterone levels among men may be related to worse cardiovascular health, there has been accumulating evidence that a normal testosterone level is, in fact, beneficial to the male cardiovascular system and that androgen deficiency is associated with adverse cardiovascular events. As testosterone falls as men age (which may in turn lead to worse cardiovascular health), emerging studies suggest that testosterone may have a future role in treating heart failure, angina, and myocardial ischemia [41]. Still, it is believed that possible cardioprotective benefits of testosterone therapy requires major research attention as evidence regarding androgen deprivation therapy remain mixed.

Health behaviors also play a dominant part role in the development of heart diseases and the excess male risk among older adults [42], including smoking, excess drinking, etc. Estimates are that around 60–80% of coronary events could have been avoided if people adhered to a low-risk lifestyle [43, 44]. Historically, men have had more adverse health behaviors such as smoking and heavy drinking, which may partially explain the gender gap in heart diseases among earlier cohorts and also the shrinking gender gap as women are adapting behaviors more similar to men [45, 46]. The impact of these risk factors appears to vary by gender. For instance, the adverse effect of smoking is more pronounced for women [47–49]. While both biology and behavior account for some of the heart disease differences between men and women, a recent study has found a higher risk of heart disease among men persisted even after all traditional risk factors are accounted for, including high cholesterol, high blood pressure, smoking, alcohol, diabetes, body mass index, and physical activity [47]. Future studies should continue to probe the underlying differences between men and women’s risk for heart disease, with a focus on the molecular and cellular physiology of the cardiovascular system.

This gender gap in heart disease is closely related to the overall excess male mortality. In the U.S., heart disease is the leading cause of death, accounting for 24.2% of all deaths for men and for 21.8% of all deaths for women in 2017 [50]. The age adjusted mortality rate for heart diseases in the US declined by more than 50% in the second half of the 20th century [51, 52]. This fall was predominantly driven by rapid declines in mortality from ischemic heart disease, as a result of progress in prevention (e.g., a decline in smoking rates among men) and evidence-based treatments (for example, surgical interventions, statins, antiplatelet agents, and antihypertensives) [53, 54]. However, the long-term reduction in heart disease mortality appears to have slowed after 2010 in many low mortality countries [55, 56]. There have even been increases in some types of heart disease mortality in the last decade in some countries including the U.S. This observed recent patterns of heart disease mortality overall may be influenced by the growing burden of cardiometabolic risk factors for heart disease. It is likely that obesity, diabetes, and hypertension play a role in the observed changes in recent mortality rates for heart disease in the US. It is projected by some that future trends of cardiovascular health may continue to worsen due to poor diet, physical inactivity/obesity, and diabetes in the coming decades [52]. It is suggested that the largest recent increases in heart failure mortality were in black men, whereas the largest increases in hypertensive heart disease occurred in white men [48]. This may be related to the end of an era of reduction in heart disease mortality fueled by reduction in smoking and increase in use of lipid lowering and hypertension control medication, which may now have reached their limit of influence. Future studies need to continue to understand the underlying mechanisms behind the excess male risk of heart failure and hypertensive heart disease as well as current trends.

Five of the 13 primarily European countries examined in Fig. 5 indicate a higher level of stroke among males than females [2]. Data from additional countries around the world have also found a higher prevalence of stroke among men which has been largely attributed to the much higher prevalence of smoking and alcohol intake among men compared to women [57]. However, there is evidence that this gender difference in stroke is partially biologically determined. For instance, estrogen is believed to have a protective effect through dilation of blood vessels and improvement of blood flow, as well as induction of anti-inflammatory factors [58]. Chromosomal influences may also play a part, as the second X chromosome is believed to raise stroke risk, even after adjusting for estrogen levels, and becomes increasingly more evident with aging [58, 59]. Men are more likely than women to die after a stroke. A meta-analysis reported that the mortality rate for those with a first stroke was significantly higher for men than women both after one year and after five years after adjusting for confounding factors, such as age, history of atrial fibrillation, pre–stroke functional limitations and stroke severity [60]. Similar findings were seen in an analysis of five international randomized controlled trials, in which men were more likely to die at 3–6 months after an ischemic stroke [61].

Like heart diseases, it appears that low testosterone level is associated with increased risk of stroke among men. A meta-analysis showed that different types of androgen deprivation therapy, such as gonadotropin-releasing hormone, oral antiandrogen, and orchiectomy, are all significantly associated with higher risk of stroke in patients with prostate cancer [62]. These findings raised awareness of the potential risks of androgen deprivation therapy and presented the need for more future studies that determine whether interventions that raise testosterone levels could prevent cerebrovascular disease in aging men.

Gender differences in the prevalence of diabetes at older ages are less consistent across countries. There is significant excess male risk of having diabetes in 5 out of the 13 countries examined (Fig. 5). It is important, first of all, to differentiate type I and type II diabetes in our discussion, as the pathological pathways of developing the two diseases are different. Type I is an autoimmune disease and results from immune cells destroying the insulin-producing pancreatic β-cells diabetes. Although the cause of Type I diabetes remains poorly understood, it believed that it is closely related to genetic susceptibility as it often occurs at a very young age [63]. A review chapter concludes that there are more than 50 genetic susceptibility regions identified to be associated with type I diabetes, including human leukocyte antigen genes, interleukin-2 pathways, cytotoxic T lymphocyte antigen 4 [64]. The prevalence of type I diabetes is higher for females until they reach puberty and then becomes more prevalent among males typically after the age of 15, which indicates that sex hormones may also play a role in the development of the disease [63]. There is some evidence that androgens are protective against type I diabetes, in which castration of male mice increases risk of the disease [65] whereas chronic androgen treatments reduce the incidence of diabetes in female mice [66]. Understanding the ability of sex hormones to influence the early development and progression of the autoimmune type I diabetes may be useful in addressing the sex differences in this disease.

Regarding type II diabetes, numerous studies have suggested that, though women may be more at risk in youth, starting in mid-life ( > = 50), the prevalence of type II diabetes tends to be higher in men than women [67, 68]. Compared to type I diabetes, type II diabetes is more prevalent in the general population and influenced by lifestyles and, in most cases, develops in later life. There are a number of gender differences in health behaviors that are believed to be related to the male excess risk in type II diabetes although note that we do not find male excess in the prevalence of diabetes in most countries. First, studies have suggested that men have less healthy nutrition by consuming more meat and fewer fruits and vegetables than women, which contributes to a higher risk of diabetes [69, 70]. Second, a meta-analysis suggests that, though active and passive smoking is related to higher risk of developing diabetes in both men and women, the overall effects tended to be even stronger in men compared to women [71]. Third, though some studies have suggested that moderate drinking is associated with risk reduction in diabetes, this association may be specific to women only, and existing data showed no sign that drinking is protective against diabetes among men [72]. Researchers believe this may be potentially explained by (1) the fact that men more frequently have worse drinking behavior with heavy episodic drinking (2) that alcohol exerts sex-dimorphic effects on glucose metabolism, (3) or that moderate alcohol consumption improved glycated hemoglobin in both sexes but tended to improve insulin sensitivity in women only [73].

Although obesity is an established risk factor, it appears that men and women have different obesity thresholds and that, on average, men who develop type II diabetes do so at a lower BMI than women [74]. This may be partially explained by the fact that men and women have different energy metabolism. At the level of the gametes, men produce sperm that are small, numerous and disposable whereas women produce large and immobile eggs; men only share genes during conception, whereas women transmit all their resources including their energy stores, their cytosol, and their mitochondria [75]. It is also important to note that, from an evolutionary perspective, female mammals resist the loss of body energy stores during long periods of food scarcity so that the offspring is not affected, while male mammals mobilize energy stores immediately for short-term muscle activity related to hunting and protection needs. These differences in the energy consumption may be directly related to the sex-specific role of fat, in which women more often store adipose tissue in subcutaneous areas that are more adapted to long-term storage, in comparison to the preferential visceral fat deposition in men that are more metabolically active and sensitive to lipolysis [76]. These evolutionary paradigms may play a part in explaining the sex differences in metabolic regulation and diabetes susceptibility, in which, despite that woman are at greater risk of obesity due to their increased propensity to gain fat, they are at lower risk of type II diabetes [75].

Several additional biological mechanisms have been suggested as a reason for excess male risk for diabetes. For instance, men may be more insulin resistant than women, which leads to increasing level of blood sugar. Compared to men of the same age, healthy women have lower skeletal muscle mass and higher adipose tissue mass, more circulating free fatty acids, and higher intramyocellular lipid content, all factors that could contribute to a more insulin-sensitive environment that helps lower blood sugar level in women compared to men [77]. Testosterone level is believed to protect against type 2 diabetes for men; testosterone deficiency has been linked to the development of visceral obesity, insulin resistance and metabolic syndrome in men [77–79]. With human genetics data becoming more available in recent years, new studies have found that a genetically higher testosterone level is associated with lower type 2 diabetes risk or lower fasting glucose in men. Interestingly, it seems that higher testosterone is beneficial in men but harmful for women, in which genetically higher level of testosterone increases the risk of type 2 diabetes for women [80]. In a number of large population-based prospective studies, low testosterone levels also predicted incident type 2 diabetes in older men [81]. All these factors are indicated as risks for diabetes; what we have observed empirically in Fig. 5 is the prevalence of diabetes, which is the result of both incidence and survival and gender specific survival may also differ.

Arthritis is more common among women in all countries examined in Fig. 5. Obesity is a risk factor common to both osteoarthritis and rheumatoid arthritis (RA), and literature has suggested that variation in the gender difference may be linked to relative national levels of obesity for men and women [82]. While arthritis is more prevalent among women as an autoimmune disease, there are some aspects of arthritis for which men have greater risk. In terms of osteoarthritis, although women are more likely to develop hand, foot, knee arthritis, the rates of shoulder and cervical spine osteoarthritis have been shown to be higher among men than women [83]. Smoking, one of the most recognized behavioral risk factors for rheumatoid arthritis (RA), is more strongly associated with RA for men than women – a meta-analysis found that male ever-smokers are at higher risk for RA development (OR: 1.89 CI: 1.56–2.28) than women (OR: 1.27; CI: 1.12–1.44) [84]. Other behavioral risk factors which have been studied in relation to the development of RA include alcohol intake, diet, and vitamin D [85–87]. However, the impact of these risk factors on both the development and severity of RA remains unclear [88].

There is strong evidence for a gender difference in depression among older adults. Women are much more likely to have depressive symptoms and higher rates of diagnosed depression than older men [89]. This lower likelihood of men having depression is shown for 13 high income countries shown in Fig. 5. Meta-analyses based on data from North America, Asia, and Europe, has concluded that the finding that older women are more likely to suffer depression is a robust and universal finding [90]. While significant research has attempted to understand this gender gap in depression, the mechanisms are complicated and remain unclear. There is some thought that part of the explanation lies in gender differences in help-seeking behavior in which women are more likely to admit and complain about their dysphoric feelings, while men are more likely to deny and instead act them out (such as through alcoholism or suicide) [91, 92]. There has also been research with a focus on biological explanations of gender difference in depression. For instance, some researchers believe that pubertal hormones, specifically estrogen, progesterone, testosterone, and adrenal androgens, may play a role in the gender gap. Meta-analyses have shown some support for this speculation with findings that gender difference in depression usually peaks in adolescence, which is possibly due to the fact that girls typically enter puberty earlier than boys and thus experience depressive symptoms along with the hormonal changes earlier [90]. However, empirical evidence for this biological explanation is scarce, especially when extended to adulthood.

Social circumstances vary for men and women and these can affect the level of depression and may be related to gender differences in the older population. One major influence on older adults’ mental wellbeing is their late-life transition into retirement. Studies have shown that the association between retirement and psychological distress is reported to be stronger among men [93]. Lee and Smith found a significant difference in depression rates in men who retired (24% for retired vs. currently employed 6%), and a somewhat smaller difference among women (29% for retired vs. 16% for currently employed) [94]. Some studies have also shown that receiving instrumental and financial support were negatively associated with mental health and more depressive symptoms in older men [95, 96]. On the other hand, women are more likely to experience the loss of a spouse as men die younger, leading to a period of bereavement and depression. Still, the association between widowhood and depressive symptoms may be stronger for men than for women, in which, over time, women may adapt to widowhood more successfully than men [97].

It is important to mention that testosterone is believed to be associated with aggressive/risk-taking behaviors (e.g., drunken driving, illegal drug use, physical fights and violence), and, in some cases, suicidal ideology [98, 99]. Sex differences in mortality are greatest among younger adults, partially because younger men are more apt to engage in risky and aggressive behaviors that generally attenuate with age [100]. Research has generally shown that men take greater physical and financial risks than women, and several studies have been able to link this difference to the high post-pubertal testosterone levels among men [101]. One study in particular showed that cortisol and testosterone shifted investment towards riskier assets. Cortisol appears to affect risk preferences directly, whereas testosterone operates by inducing increased optimism about future price changes [102]. It is suggested that the balance between testosterone and cortisol may be predictive of both impulsive and instrumental aggression, and one study shows that acutely increasing testosterone potentiates aggressive behavior in men but only among those with dominant or impulsive personality styles [103]. Lastly, though it is traditionally believed that there is a link between androgens and suicidality, results have been inconsistent. A recent longitudinal study that followed the same group of people over the course of 9-year found that androgen levels were not significantly associated with future suicidal ideation or attempts [104]. In fact, one study showed that there is an increased risk of suicide in patients undergoing androgen deprivation therapy, even after controlling for confounding factors such as severity of comorbidities and age [105].

Finally, regarding cognitive differences, it is generally agreed that women are more likely to have dementia than men. While this gender gap is partially explained by women’s longer life expectancy, some researchers suggested that women decline to dementia at a faster pace compared to men due to both biological and social differences [106]. Biologically, the E4 allele of the apolipoprotein E gene, the strongest known susceptibility variant for AD, has a stronger effect on women than men [107]. Socially, some of the female disadvantage in cognitive function in many countries may be accounted for by education, as historically in many countries women have had less access to education in earlier cohorts [108]. Still, in terms of executive function and global cognition, women experience faster decline, a disadvantage that persists even after adjusting for education, leaving room for additional contributors and biological pathways that play a role [109]. Women’s higher risk of depression may also partly account for the gender gap, as psychological distress is typically associated with cognitive impairment [110].

## Physiological deterioration

**Fig. 6:**
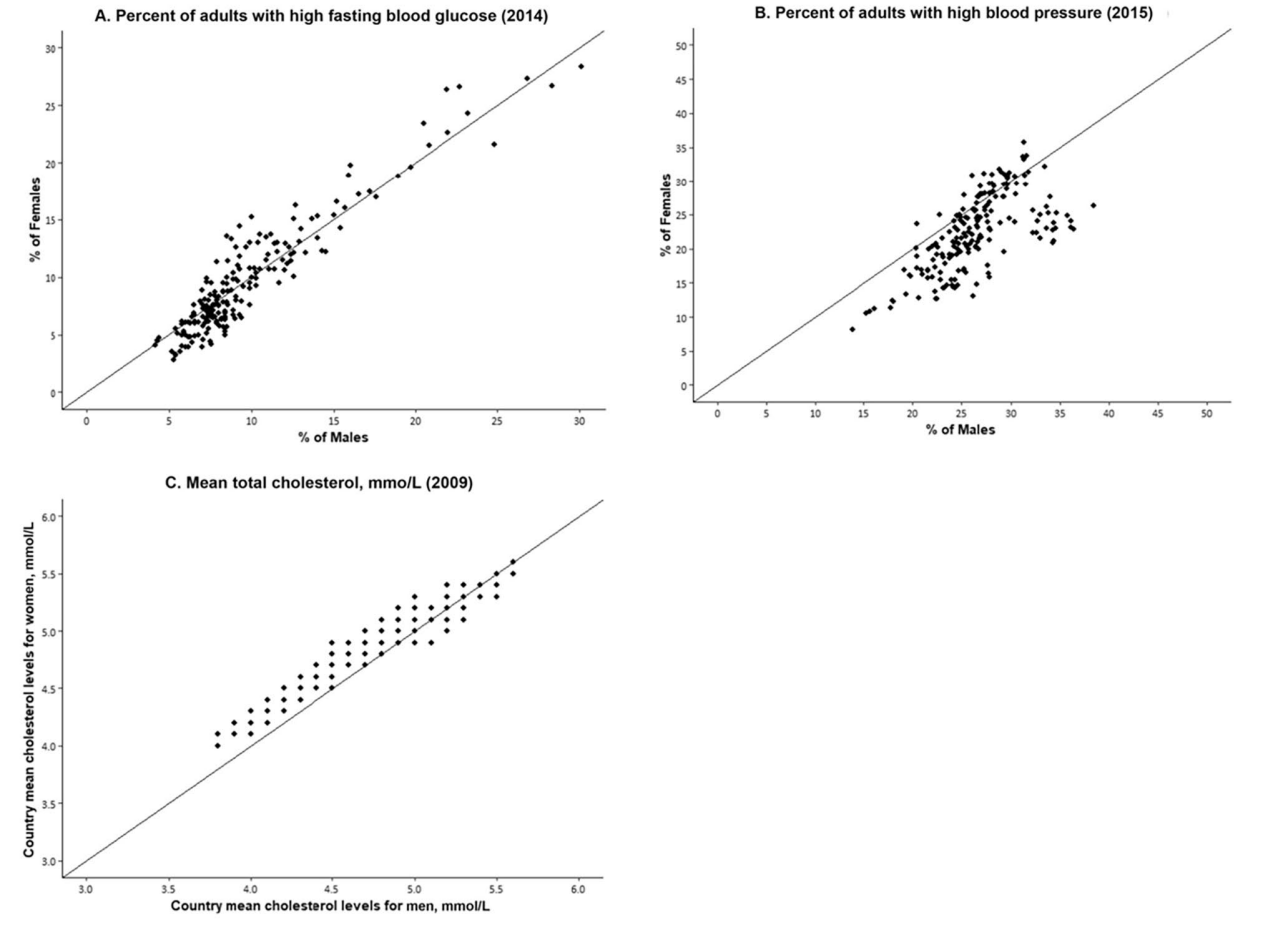
Percent of Men/Women with High Risk Levels of Fasting Glucose and High Blood Pressure and Mean Total Cholesterol in 191 Individual Countries. Original data from WHO Global Health Observatory Data Repository [111]

**Fig. 7:**
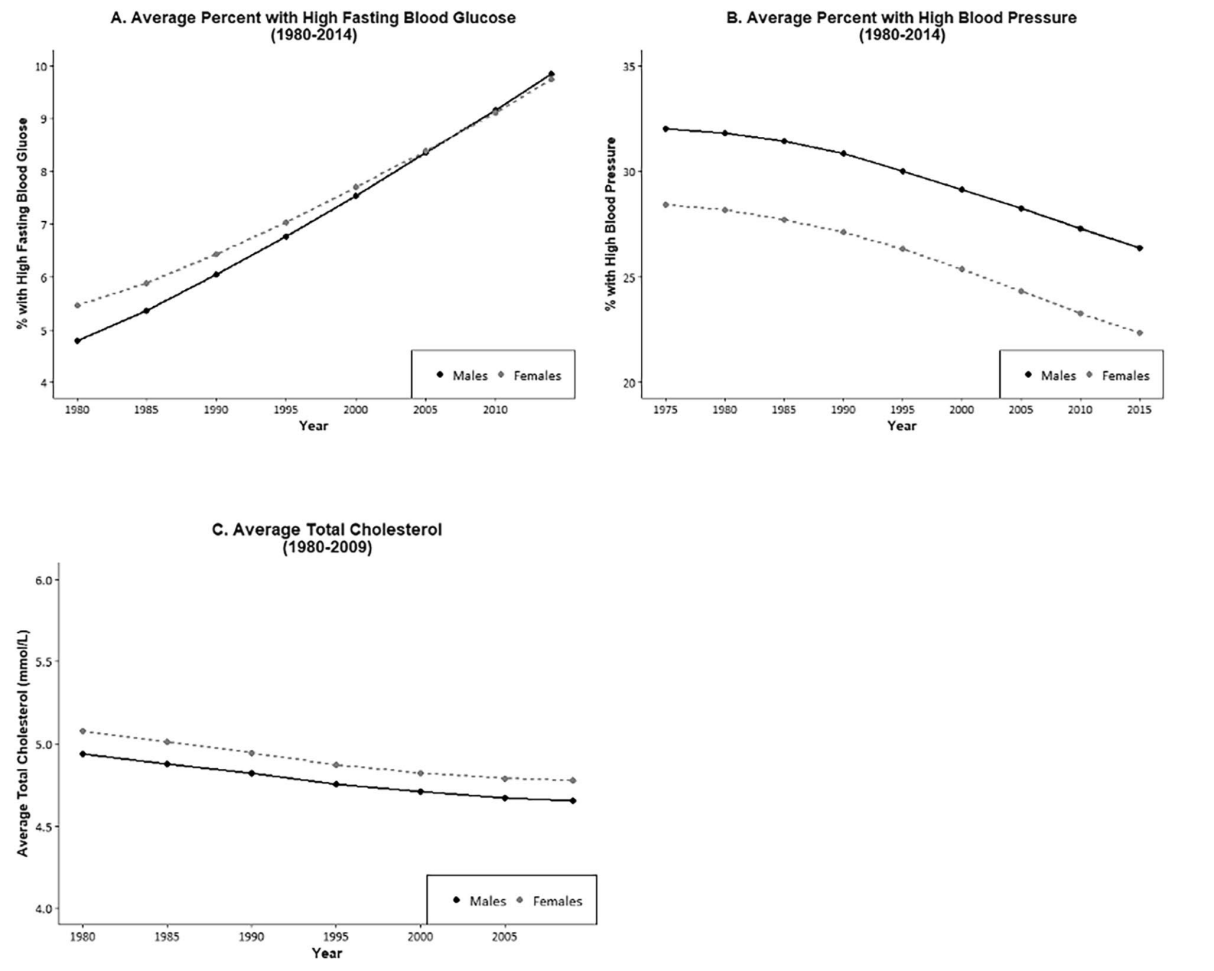
Average Percent of Men and Women with High Glucose, High Blood Pressure, and Average Cholesterol Level in almost 200 Countries over Recent Decades. The number of countries is 191 for blood glucose and blood pressure, and 189 countries for cholesterol. Values are age standardized. Data are from WHO Global Health Observatory Data Repository [111]

We now examine sex differences in indicators of physiological status that are recognized risk factors for the development of diseases, mortality, and aging. Examining those indicators by sex may help to clarify the mechanisms behind the sex differentials in downstream dimensions of health and mortality. Figures 6 and 7 show WHO data for 191 countries on the prevalence and trends in risk levels for glucose, hypertension, and cholesterol by sex [111]. First, Fig. 6 shows that, within countries, the national prevalence of high glucose is not clearly higher for men or women in 2014. In terms of time trends (Fig. 7), the average percent of adults with high glucose has been steadily growing for both men and women in the past 3 decades; however, the percent appears to be growing faster for men than women. Though men used to have a lower prevalence of high glucose compared to women, men increased to the level of women by 2005 and then continued to grow at a slightly faster rate.

On the other hand, the prevalence of high blood pressure for men exceeds that for women in most countries, although there are some countries where it was higher for women in 2015 (Fig. 6). In the past 4 decades, there has been consistent and impressive decline in the prevalence of high blood pressure prevalence around the globe, although the excess male risk remains relatively constant over time (Fig. 7).

The sex differential in total cholesterol appears to depend on the national level of cholesterol. Women have higher mean values of cholesterol at lower levels of cholesterol; at higher national levels of cholesterol, men are more likely to have higher levels. The average cholesterol level across countries has declined in the past several decades and the decline has been similar for men and women so the differential has not changed.

It is not only the level of these risk factors that differs by gender, but the impact of these physiological risk factors on heart diseases appears to vary by gender. For instance, regarding the risk of myocardial infarction, while the adverse effect of high blood pressure is more pronounced for women, the adverse effect of high total cholesterol is more pronounced for men [40, 42, 43].

**Fig. 8:**
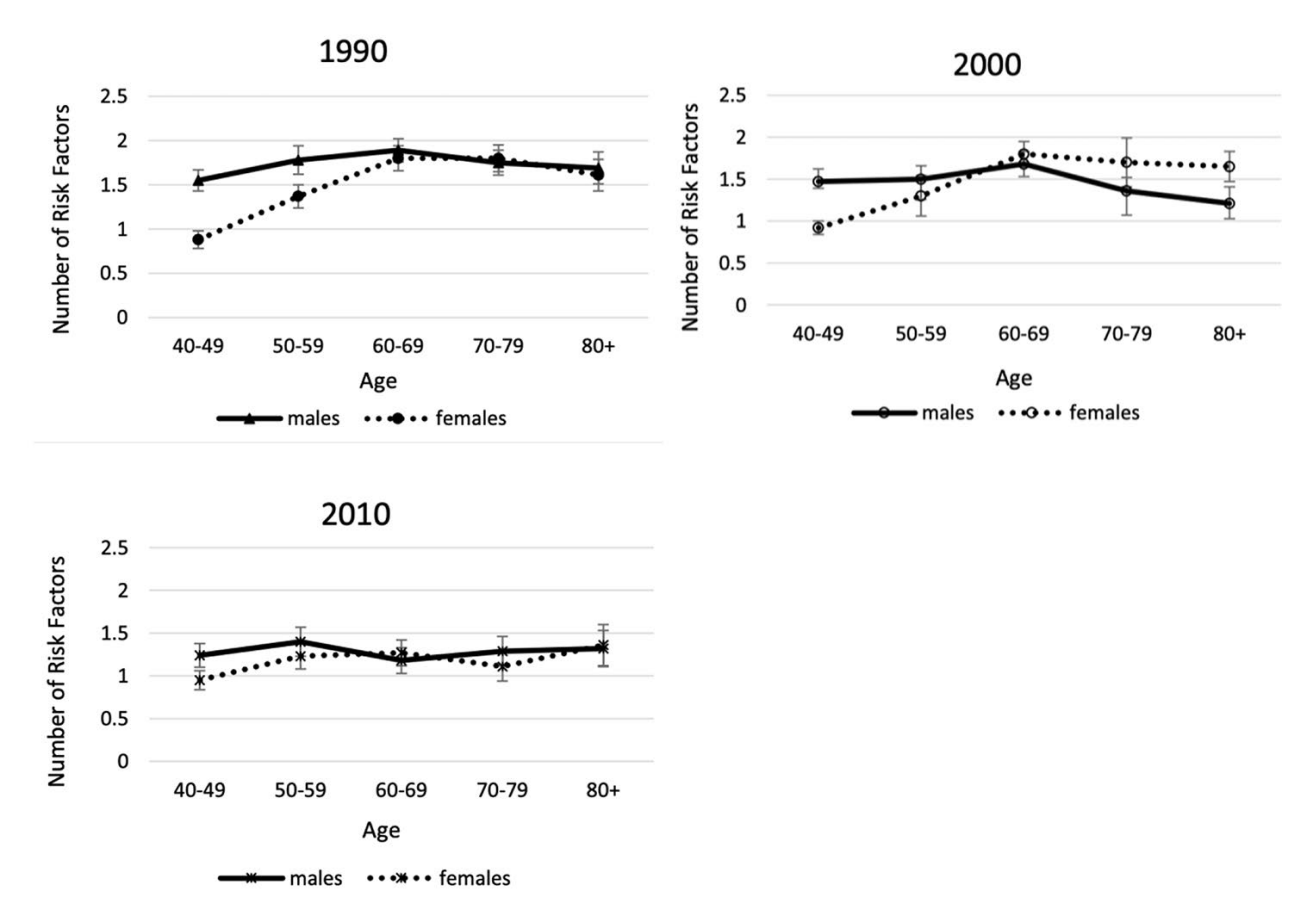
**Mean number of high-risk cardiovascular risk factors (range: 0–8) by age and sex among men and women aged 40 and over in the United States: 1990, 2000, 2010**. Data are from NHANES. Cardiovascular risk indicators include systolic and diastolic blood pressure, body mass index (BMI), total cholesterol, high-density lipoprotein (HDL) cholesterol, low density lipoprotein (LDL) cholesterol, triglycerides, and glycated hemoglobin (HbA1c) [112]

Time change in overall cardiometabolic risk which summarizes multiple risk factors for American men and women provides a good example of the fact that sex differences are not constant over time in any one country. Kim et al. examined sex differences in a summary indicator of cardiometabolic risk, which includes at-risk levels of systolic and diastolic blood pressure, body mass index (BMI), total cholesterol, high-density lipoprotein (HDL) cholesterol, low-density lipoprotein (LDL) cholesterol, triglycerides, and glycated hemoglobin (HbA1c) from 1990 to 2010 for people over age 40 [112]. Their results showed that men had higher mean age-specific cardiovascular risk up to age 60 in 1990, and after age 60 the sexes had similar risk (Fig. 8). In 2000, men had excess risk up to age 60; and after that age, women had excess risk (Fig. 8). By 2010, there were no sex differences in cardiometabolic risk over the age of 50. This growing similarity over time in cardiometabolic risk of men and women represents a remarkable shift from our conventional understanding of risk profiles associated with sex. Kim et al. believe that the substantially greater reduction in the risk of having high systolic blood pressure from 2000 to 2010 among women contributed to this. While both men and women had an increase in blood pressure management medication and improvement in control of blood pressure, the increase in effectiveness of medication in controlling systolic blood pressure appeared to be more effective among women, resulting in a larger improvement of women’s systolic blood pressure in the recent decade.

## Molecular and Cellular Aging

**Fig. 9:**
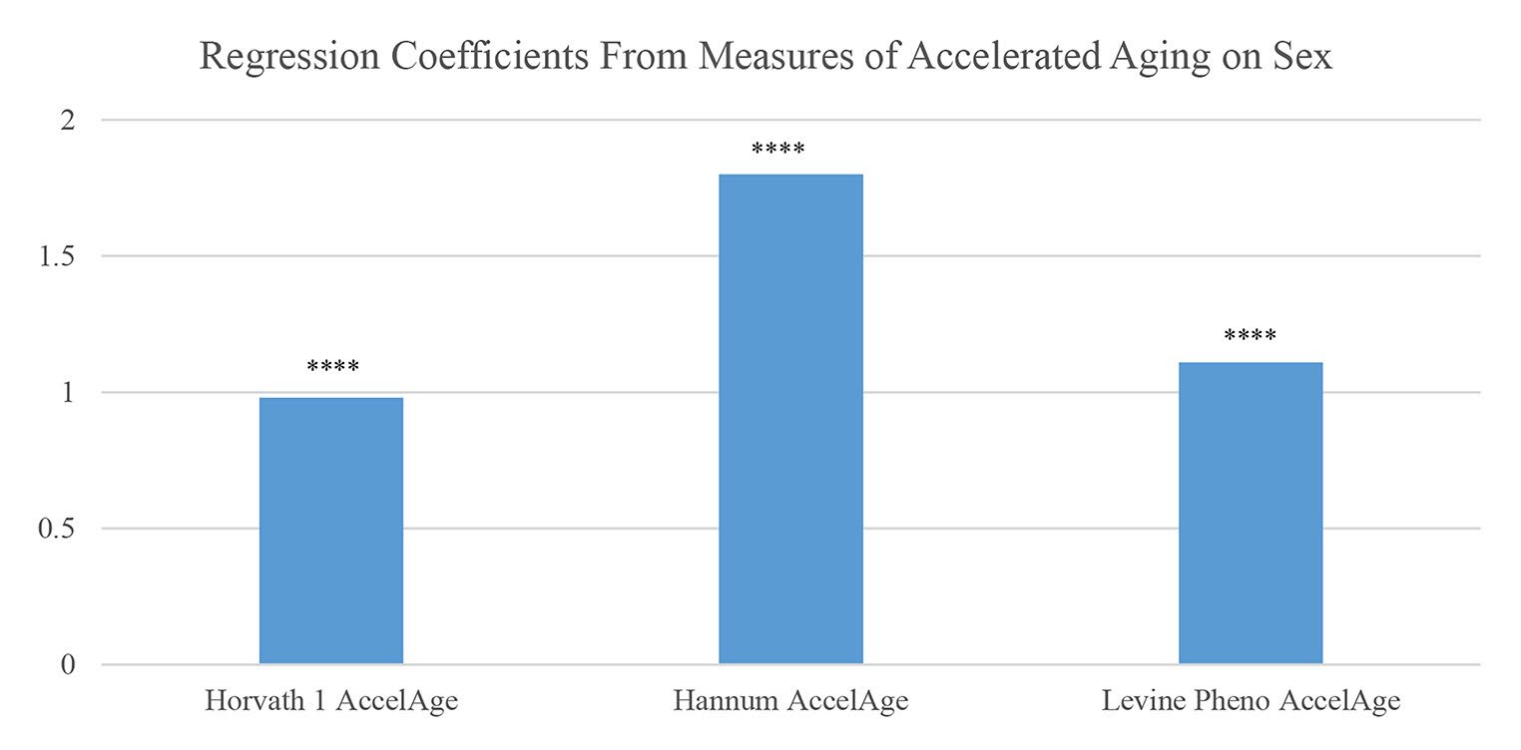
Years of accelerated epigenetic aging and accelerated pace of aging for men compared to women based on 3 epigenetic clocks: Americans over age 56. Notes: Controlled for education, race/ethnicity, obesity, smoking, and cell distribution based on HRS flow cytometry. Source: Data are from the Health and Retirement Study [114]

Recent research has begun to explore sex differences in the Hallmarks of Aging [113]. These are molecular and cellular mechanisms thought to underlie the entire process of health change with age; as such, they are assumed to contribute to development of all the downstream dimensions of morbidity as well as mortality. While empirical work on human populations is just beginning, the markers that can be measured in population studies generally indicate accelerated aging for men relative to women. Epigenetic changes are one hallmark of aging and men have accelerated epigenetic age relative to women based on DNA methylation as indicated in three epigenetic clocks (Fig. 9) [114]. The figure indicates that men’s epigenetic ages exceed those of women by estimates of about one to two years.

**Fig. 10:**
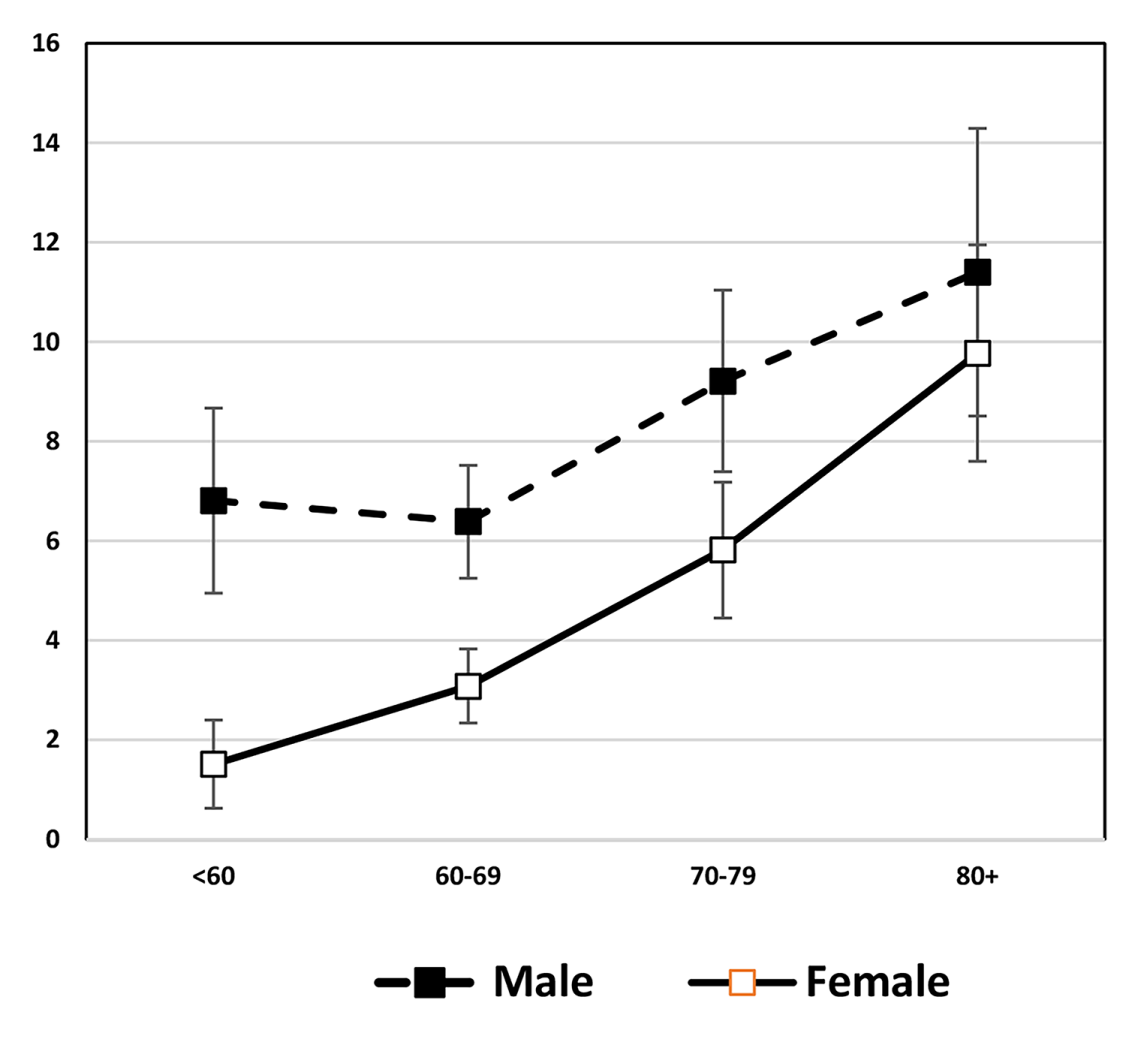
Percent Immunosenescent (% CD4/CD8 < 1) by age and sex among Americans over age 56: Health and Retirement Study. 2016 Data from Health and Retirement Study [115]

Men also appear to have greater senescence of immune function with aging, another indicator of overall aging, as shown in the proportion of men over the age of 56 who have a low ratio of CD4/CD8 T cells (Fig. 10) [115]. There has been some evidence that there may be a trade-off between immune functioning and testosterone-dependent sexual signals. High testosterone levels may compromise the balance between the production of reactive oxygen species, which is produced to kill internalized pathogens, and their elimination by antioxidant defenses, leading to a possible increased risk of oxidative stress, which is believed to be involved in several age-related morbidities such as cardiovascular diseases, chronic obstructive pulmonary disease, chronic kidney disease, neurodegenerative diseases, etc. [116, 117].

It has also been reported that men have shorter telomeres than women [118], indicating more rapid aging. In addition, men were reported to have significantly lower mitochondrial DNA than women, which may be due to differing levels of white blood cells [119] and estrogen levels [120]. These factors while assumed to indicate the basic biology of aging are all influenced by lifestyle factors (e.g., higher smoking exposure in men) as well as basic biology [121]. So while all of these differences may be pathways influencing disease, functioning, and mortality as well as downstream physiological differences between the sexes, they reflect both biology and lifetime exposures and circumstances that differ by sex.

A significant amount of work has incorporated genetic risk into analysis of health outcomes examined so far – i.e., disease, mortality, cognitive loss. A relatively recent approach has been to develop polygenic risk scores (PRS) based on GWAS results from multiple studies of large populations. PRS scores have been computed for numerous health outcomes. An examination of sex differences in 14 PRS for a large nationally representative cohort of older Americans indicated that only the score for Schizophrenia differed by sex, with females showing more genetic risk [122]. This does not capture the effect of sex chromosomes but indicates that the identified genetic risks for disease and mortality do not differ by sex.

It is important to include antagonistic pleiotropy (AP) in this discussion. Antagonistic pleiotropy suggests that the same genes may produce positive effects in one context but be deleterious in another context. For instance, some genes can be beneficial early in life as they promote growth and sexual reproduction, whereas, in older adulthood, the same genes may promote certain diseases and have detrimental effects on health. Similarly, it is also believed that some genes may elevate fitness in one sex but be harmful to the other; or they could be detrimental to each sex in different ways [123]. One of the only genes with empirical evidence for this is p53, a gene that regulates apoptosis and metabolism and is mutated in the majority of human cancers. P53 has sex-specific effects on the life spans of females and male drosophila, in which wide-type p53 over-expression limits life span in females and favors life span in males whereas null mutation of the endogenous p53 gene increases life span in females and has smaller effects on male life span [124]. These findings may have implications for human aging related diseases (e.g., cancer), in which the effects of human p53 and p53-interacting genes on cancer incidence and longevity are also often sexually dimorphic [125, 126]. Future studies are needed to continue identify specific pleiotropic loci that act antagonistically within or between sexes and explore how those AP loci may be used to explain or modify sex differences in diseases that are believed to be related to AP, such as cancer and neurodegenerative diseases [125].

## Conclusions

This analysis of sex differences in health used five dimensions of the morbidity process (Fig. 1), which categorized multiple health indicators according to the process of health change with age at the population level. Our aim is to investigate how these dimensions of health differ for men and women, and whether the sex differences were similar across historical time and between countries. Overall, our analysis suggests, although the sex differences in all dimensions of health are quite complex and not fixed over time and place but affected by biology that is very responsive to behaviors as well as the epidemiological environment.

First, regarding mortality, although it has been established that male life expectancy is now lower than female life expectancy in all countries, there is clear variability in the size of the differential due to the behavioral and epidemiological differences between men and women across regions and time. As infectious disease mortality was largely replaced by chronic disease mortality, such as cardiovascular conditions and cancers, the change in the relative level of mortality rates for men and women becomes closely associated with risk-related behaviors (e.g., obesity, cigarette smoking, alcohol consumption) and management of those conditions. With an increase in cardiometabolic risk factors in some developed and developing countries in recent years, there is a need to better promote healthy lifestyles (e.g., better diet and physical activity) and provide better preventive care and health screening for both men and women. There is some evidence that as men and women behave more similarly in their behaviors related to cardiometabolic risk, their health outcomes are more similar. As the gender gap in mortality is also partially biologically determined, which is reflected in the higher male mortality in infancy and decreased life expectancy among heterogametic species (including male humans), there is also a need for further understanding on the mechanisms of how sex chromosomes and hormones influence longevity directly as well as through behavior in order to assess potential interventions.

Secondly, regarding morbidity, our analysis suggests that, on average, sex differences in the prevalence of diseases and conditions generally indicate that men have a higher prevalence of more lethal conditions (heart disease and stroke), whereas women are more likely to have chronic but non-fatal diseases and conditions (arthritis, depressive symptoms and disability), though there is considerable variation between countries in the difference between the sexes, which, similar to mortality, may be explained by behavioral and social differences between women and men. For example, men are more likely to eat more meat and fewer vegetables, smoke, and drink excessively in many countries, which are potential risk factors for heart diseases, stroke and diabetes. Behaviors or social conditions that are more common among women, such as physical inactivity and higher likelihood of losing a spouse, may help explain their excess risk in functional limitation and depression. Nevertheless, men and women have different biological predispositions to morbidity, and these biological predispositions interact with behaviors (e.g., obesity and drinking) to produce observed differences in diseases and conditions. A repeated theme in our review is the effect of the testosterone level and as men’s testosterone level decreases with age, some researchers have called for further understanding of the feasibility of testosterone therapy in treating various cardiovascular related conditions as current evidence remain mixed. On the other hand, natural estrogen appears to be protective of heart diseases and stroke among women but supplementation with estrogen which was once very common is now not recommended for prevention of cardiovascular disease [127, 128]. In the future, further research and understanding of the role these sex hormones play may be critical in health interventions to address gender gaps in diseases and mortality.

There are numerous candidates for explanation of why men and women differ in their length of life and in health problems: sex hormones, inflammatory responses, behaviors, cultural factors and it is impossible to disentangle them. First, the shorter lives of men and the higher risk for lethal conditions may reflect differences in sex hormones with women’s sex hormones being protective and men’s providing more risk in this epidemiological environment [129]. This is clearly only a part of any story, however, because the size of the sex difference in the length of life and most disease prevalences varies across countries and over time. Even the influence of sex hormones must interact with broader social and epidemiological circumstances to result in the observed differences. Secondly, inflammation may be both a basic mechanism of health change with age and, in the modern world where infection no longer accounts for many deaths, women’s more responsive immune systems may be a factor in their longer lives as well as their worse functioning and high prevalence of immune related disorders relative to men. Women’s immune systems may have been developed to ensure survival of the species in a time of low life expectancy and high infectious mortality. The cost of the related higher levels of arthritis and other autoimmune conditions now occurring among women was not as great when life expectancy was shorter. Gendered differences in immune functioning may have been partly responsible for reduced male ability to fight off a novel infection as seen in the ongoing COVID pandemic. Thirdly, regarding behaviors, the changes we have seen recently in cardiovascular risk among American men and women indicate the responsiveness of sex differences to behavior change as well as medical intervention. These changes may operate on basic differences in biology but significant changes over recent decades in male/female differences in mortality as well as disease indicate that where men and women are more alike in their behaviors, education, and medical intervention, they are more alike in their health outcomes.

The future of male and female differences in morbidity and mortality should be reflected in the differences in very most basic measures of molecular and cellular aging; however, it is difficult to statistically quantify at this point how these mechanisms relate to sex and the observed sex differences in population health. Future research is required to further our understanding of the link between sex and the process of molecular and cellular aging that produces disease, loss of functioning, and mortality. In addition, we need to focus research on how these biological factors interact with behaviors, social factors and the epidemiological environment that is going to continue to change in the future.

## Abbreviations

ADL: Activities of daily living
IADL: Instrumental activities of daily living
OR: Odds ratio
RA: Rheumatoid Arthritis
PRS: Polygenic risk score
